# HIV-Specific Antibody-Dependent Cellular Cytotoxicity (ADCC) -Mediating Antibodies Decline while NK Cell Function Increases during Antiretroviral Therapy (ART)

**DOI:** 10.1371/journal.pone.0145249

**Published:** 2015-12-22

**Authors:** Sanne Skov Jensen, Anders Fomsgaard, Marie Borggren, Jeanette Linnea Tingstedt, Jan Gerstoft, Gitte Kronborg, Line Dahlerup Rasmussen, Court Pedersen, Ingrid Karlsson

**Affiliations:** 1 Virus Research & Development Laboratory, Department of Microbial Diagnostic and Virology, Statens Serum Institut, Copenhagen, Denmark; 2 Department of Infectious Diseases, Odense University Hospital, DK-5000 Odense, Denmark; 3 Infectious Disease Research Unit, Clinical Institute, University of Southern Denmark, Odense, Denmark; 4 Viro-immunology Research Unit, Department of Infectious Diseases, Copenhagen University Hospital, Copenhagen, Denmark; 5 Department of Infectious Diseases, Copenhagen University Hospital, Hvidovre, Denmark; Institute of Infection and Global Health, UNITED KINGDOM

## Abstract

Understanding alterations in HIV-specific immune responses during antiretroviral therapy (ART), such as antibody-dependent cellular cytotoxicity (ADCC), is important in the development of novel strategies to control HIV-1 infection. This study included 53 HIV-1 positive individuals. We evaluated the ability of effector cells and antibodies to mediate ADCC separately and in combination using the ADCC-PanToxiLux assay. The ability of the peripheral blood mononuclear cells (PBMCs) to mediate ADCC was significantly higher in individuals who had been treated with ART before seroconversion, compared to the individuals initiating ART at a low CD4^+^ T cell count (<350 cells/μl blood) and the ART-naïve individuals. The frequency of CD16 expressing natural killer (NK) cells correlated with both the duration of ART and Granzyme B (GzB) activity. In contrast, the plasma titer of antibodies mediating ADCC declined during ART. These findings suggest improved cytotoxic function of the NK cells if initiating ART early during infection, while the levels of ADCC mediating antibodies declined during ART.

## Introduction

Antiretroviral therapy (ART) significantly reduces HIV-related morbidity and mortality [1]. The early initiation of ART reduces the rates of transmission of HIV [2] and improves clinical benefit for HIV infected individuals [3, 4]. Despite the obvious benefits of ART, the ideal solution would be to develop HIV-1 vaccines that either induce protective immunity or modulate immunity against HIV to control viremia in the absence of ART [5]. It has been shown that HIV-1 vaccines can induce antibodies that bind to HIV infected cells and mediate antibody-dependent cellular cytotoxicity (ADCC) [6–10]. A greater understanding of ADCC during ART is important in the development of novel strategies to control HIV-1 infection.

It has been shown that reducing the HIV viral load with ART partially restores lytic activity [11] and natural killer (NK) cell-mediated killing [12]. Only a few studies have investigated the effects of ART on ADCC mediating antibodies [13, 14] and the effector cells mediating ADCC [13]. ADCC occurs when FcγIIIa (CD16) receptors expressed on the NK cells bind to the Fc portion of immunoglobulin G (IgG) antibodies, which are bound to HIV envelope epitopes on infected cells [15–17]. NK cells are often divided into CD56^neg^ and CD56^pos^ subsets. The dysfunctional CD56^neg^ NK cell population is significantly less cytolytic and secretes lower levels of cytokines compared to the CD56^pos^ NK cells [18]. The CD56^pos^ NK cells are often divided into the cytolytic CD56^dim^ and the cytokine-secreting CD56^bright^ subsets [19].

Different NK cell markers have been identified and can be used to investigate NK cell development, subsets and function [20]. CCR7, CD27, CD57 and CD70 are known to be up-regulated [21–26] during HIV infection, while NKp46 is down-regulated during HIV infection [27, 28].

In this study, we compared peripheral blood mononuclear (PBM) effector cell cytotoxicity, NK cell phenotype and subset distribution, and ADCC mediating antibodies between ART-naïve individuals and individuals who initiated ART at different stages of HIV-disease. Individuals, who received ART, had initiated treatment either prior to seroconversion, when CD4^+^ T cell counts was above 350 cells/μl blood or when CD4^+^ T cell counts were below 350 cells/μl blood. This study demonstrates a connection between the time of initiation of ART and the ability of NK cells to mediate ADCC, which may be explained by changes in NK cell subset distribution and NK cell phenotype.

## Study Participants and Methods

### Ethics statement

The study was approved by the National Committee for Health Research Ethics of the Danish Ministry of Health (H-3-2011-031 and H-3-2012-104). All study participants provided written informed consent.

### Study participants

A total of 53 HIV-1 positive individuals followed at the University Hospitals of Copenhagen, Rigshospitalet and Hvidovre, or Odense University Hospital in Denmark were recruited to the study. Eight individuals were treated before seroconversion, 9 individuals had started ART with a CD4^+^ T cell count above 350 cells/μl blood, 18 individuals had started ART with a CD4^+^ T cell count below 350 cells/μl blood at a relevant immunodeficiency, and 18 individuals were ART-naïve. Participants on ART were viral suppressed for at least two years before sampling. Table 1 outlines the clinical characteristics of study participants.

**Table 1:** Clinical characteristics of study participants.

Group	Study ID	Sex	Age, years	Duration of ART, years	Treatment at sampling time	HIV-1 subtype	CD4^+^ T cells count at ART initiation, cells/μl	CD4^+^ T cell count, cells/μl	Lymphocyte number, *10^9^/L	HIV-1 plasma viral load, copies/ml	Plasma CRP, μg/ml	Plasma IL-6, pg/ml
ART before sero conversion, n = 8	3	m	69	8	Truvada, Norvir, Prezista	B	460	940	2	19	0.97	2.93
	7	m	40	5	Kivexa, Stocrin	B	238	696	2.9	<20	0.69	< 0.78
	8	m	30	5	Epivir, Viread, Viramune	B	570	1500	4.09	19	6.3	< 0,78
	13	m	29	2	Atripla	B	520	950	3.05	79	3.6	< 0.78
	21	m	45	6	Atripla	B	320	1100	2.6	19	0.28	< 0.78
	22	m	35	5	Kivexa, Norvir, Reyataz	B	530	900	2.29	19	1.03	< 0.78
	65	m	58	9	Atripla	B	412	994	2	<20	2.22	< 0.78
	66	m	38	5	Atripla	B	490	521	2	<20	0.15	< 0.78
Median (IQR)	-	-	39(31–55)	5(5–7.5)	-	-	475(343–528)	945(747–1074)	2.4(2.00–3.01)	19.5(19–20)^1^	1.00(0.38–3.26)	0.78(0.78–0.78)^2^
ART at CD4^+^ T cell count >350 cells/μl, n = 9	11	m	39	4	Truvada, Viramune	B	630	950	1.6	20	3.02	< 0.78
	18	m	37	3	Truvada, Isentress	B	520	530	1.24	19	0.1	< 0.78
	25	m	28	6	Kivexa, Stocrin, Sustiva	B	500	680	1.6	20	0.85	< 0.78
	27	m	39	4	Atripla	B	350	710	2	19	0.37	< 0.78
	28	m	41	4	Truvada, Viramune	B	390	660	1.8	19	0.86	< 0.78
	73	m	64	5	Truvada, Prezista, Norvir	B	418	269	1.2	53	9.17	< 0.78
	80	m	43	2	Atripla	B	492	1170	2.2	NA	1.75	4.4
	83	m	45	2	Atripla	A	492	575	NA	20	0.91	12.5
	122	m	66	16	Tivicay, Truvada	NA	360	670	1.63	<20	2.34	< 0.78
Median (IQR)	-	-	41(38–55)	4(2.5–5.5)	-	-	492(375–510)	670(553–830)	1.6(1.33–1.95)	20(19–20)^1^	0.91(0.61–2.68)	0.78(0.78–2.59)^2^
ART at CD4^+^ T cell count <350 cells/μl, n = 18	5	m	39	6	Atripla	B	160	510	2.5	<20	1.32	3.08
	15	f	24	2	Truvada, Norvir, Reyataz	D	330	890	2.1	19	0.4	< 0.78
	16	m	58	3	Atripla	B	200	560	2.3	19	10.15	1.25
	17	m	40	5	Epivir, Viread, Stocrin, Sustiva	B	180	520	2.17	39	0.74	0.98
	31	m	41	4	Truvada, Viramune	B	150	750	2.23	19	8.02	< 0.78
	32	f	65	7	Epivir, Norvir, Prezista	C	250	360	1.6	19	0.63	< 0.78
	35	m	44	4	Truvada, Isentress	B	310	520	2.7	19	0.31	1.05
	62	m	50	5	Atripla	B	40	513	1.8	20	2.67	< 0.78
	89	m	30	10	Atripla	B	190	757	2.2	<20	20.99	2.83
	90	m	51	8	Atripla	B	105	885	2.4	<20	7.46	3.41
	92	m	38	5	Norvir, Prezista, Viread, Epivir	B	157	1240	3.8	28	7.07	5.67
	94	m	69	8	Atripla	B	197	565	1.4	<20	0.91	1.62
	96	f	32	8	Atripla	G	188	446	1.8	912	0.69	1.12
	97	m	59	4	Atripla	B	193	756	2.9	20	2.28	2.73
	98	f	47	5	Atripla	B	199	504	1.9	20	3.83	3.75
	118	f	67	17	Norvir, Reyataz, Ziagen, Viread, Viramune	B	50	570	2.28	<20	0.32	<0.78
	119	m	68	16	Truvada, Isentress	NA	260	610	1.94	<20	0.42	<0.78
	120	m	59	18	Kivexa, Tivicay	NA	180	670	2.5	<20	0.89	<0.78
Median (IQR)		-	49(39–61)	5(4–8.5)	-	-	189(155–213)	568(512–756)	2.2(1.88–2.50)	20(19–20)1	1.11(0.58–7.17)	1.09(0.78–2.89)2
ART- naïve, n = 18	9	m	43	-	-	B	-	620	2.94	5,687	0.55	<0.78
	10	m	46	-	-	AE	-	530	NA	414,911	3.77	<0.78
	36	m	34	-	-	B	-	570	2.6	7,737	0.86	<0.78
	38	m	36	-	-	B	-	1100	3.8	1,600	2.46	1.79
	41	m	48	-	-	B	-	1200	3.8	40,996	4.81	<0.78
	45	m	42	-	-	NA	-	710	2	231	23.25	<0.78
	46	m	51	-	-	AE	-	860	1.64	180	3	<0.78
	48	m	49	-	-	B	-	1100	4.2	11,747	2.66	<0.78
	110	m	47	-	-	NA	-	510	1.6	43	4.05	1.74
	113	f	54	-	-	NA	-	862	1.9	298	5.31	3.22
	121	m	43	-	-	B	-	640	4.9	29,243	1.62	<0.78
	123	m	57	-	-	NA	-	500	2.08	33,001	NA	NA
	PV10	m	42	-	-	B	-	660	NA	53,108	NA	NA
	PV20	m	38	-	-	B	-	497	NA	181,000	NA	NA
	PV22	m	35	-	-	B	-	579	NA	22,000	NA	NA
	PV24	m	NA	-	-	B	-	1124	NA	17,400	NA	NA
	PV26	m	49	-	-	B	-	399	NA	1,830	NA	NA
	PV27	m	43	-	-	B	-	513	NA	46,000	NA	NA
Median (IQR)	-	-	43(40–49)		-	-	-	630(512–922)	2.6(1.90–3.80)	14,574(1275–42247)	3.00(1.62–4.81)	0.78(0.78–1.74)^2^

Female (f); Male (m); Not available (NA); Interquartile Range (IQR).

^1^When viral load was reported as viral load<20 copies/ml, 20 was used for calculating medians and IQR.

^2^The lower detection limit of the Il-6 is <0.78 μg/ml. Therefore, 0.78 μg/ml was used for calculations of medians and IQR.

The HIV-negative plasma and cells were obtained from HIV-negative individuals enrolled in the same study. The buffy coats from two HIV-negative individuals were obtained from the Danish blood bank. The peripheral blood mononuclear cells (PBMCs) were obtained by density-gradient centrifugation and were cryopreserved until the analysis was performed.

### Clinical parameters for study participants

The plasma viral loads were quantified using the COBAS Ampliprep/COBAS TaqManHIV-1 Test, version 2.0 system (Roche Diagnostics, Copenhagen, Denmark). The absolute CD4^+^ T cell counts were determined using the FACS Count system (BD Bioscience, San Jose, CA) according to the manufacturer’s protocol. The high-sensitivity C-reactive protein (CRP) levels were determined using a Brahms CRPus Kryptor (Thermo Scientific, Henningsdorf, Germany) according to the manufacturer’s protocol. IL-6 was determined using a Quantikine HS kit for Human IL-6 (R&D system, Minneapolis, USA) according to manufacturer’s instructions.

### ADCC assay

The ADCC assay was performed using PanToxiLux (OncoImmunin, Gaithersburg, MD) as described for the GranToxiLux assay [13, 29]. Cryopreserved PBMCs from HIV-positive or -negative individuals were used as the source of the NK cells. When testing individuals’ plasma, the same two HIV-negative donors were pooled and used throughout the study. CEM.NKR_CCR5_ cells [30] were coated with recombinant gp120 (0.01 mg/ml) or gp140 (0.02 mg/ml) and used as target cells. Recombinant gp120 HIV-1 representing the envelopes of subtype B, BaL (Immune Technology, New York, NY) and subtype AE, CM243 (Science Protein, Meiden, CT) were used. Moreover, recombinant gp140 HIV-1 representing the envelope of subtype A, UG37, (Polymun Scientific, Klosterneuburg, Austria) was used. When testing the ADCC activity of the plasma, the protein used for coating was, if possible, matched to the individuals’ HIV-1 subtype (Table 1). Plasma from individuals with either an unknown HIV-1 subtype or with either subtype G or C was tested against subtype B. Individuals’ PBMCs was tested together with HIV IgG immunoglobulin (HIVIG) obtained from the NIH AIDS Research and Reagent Program and was always tested against subtype B.

**Fig 1:**
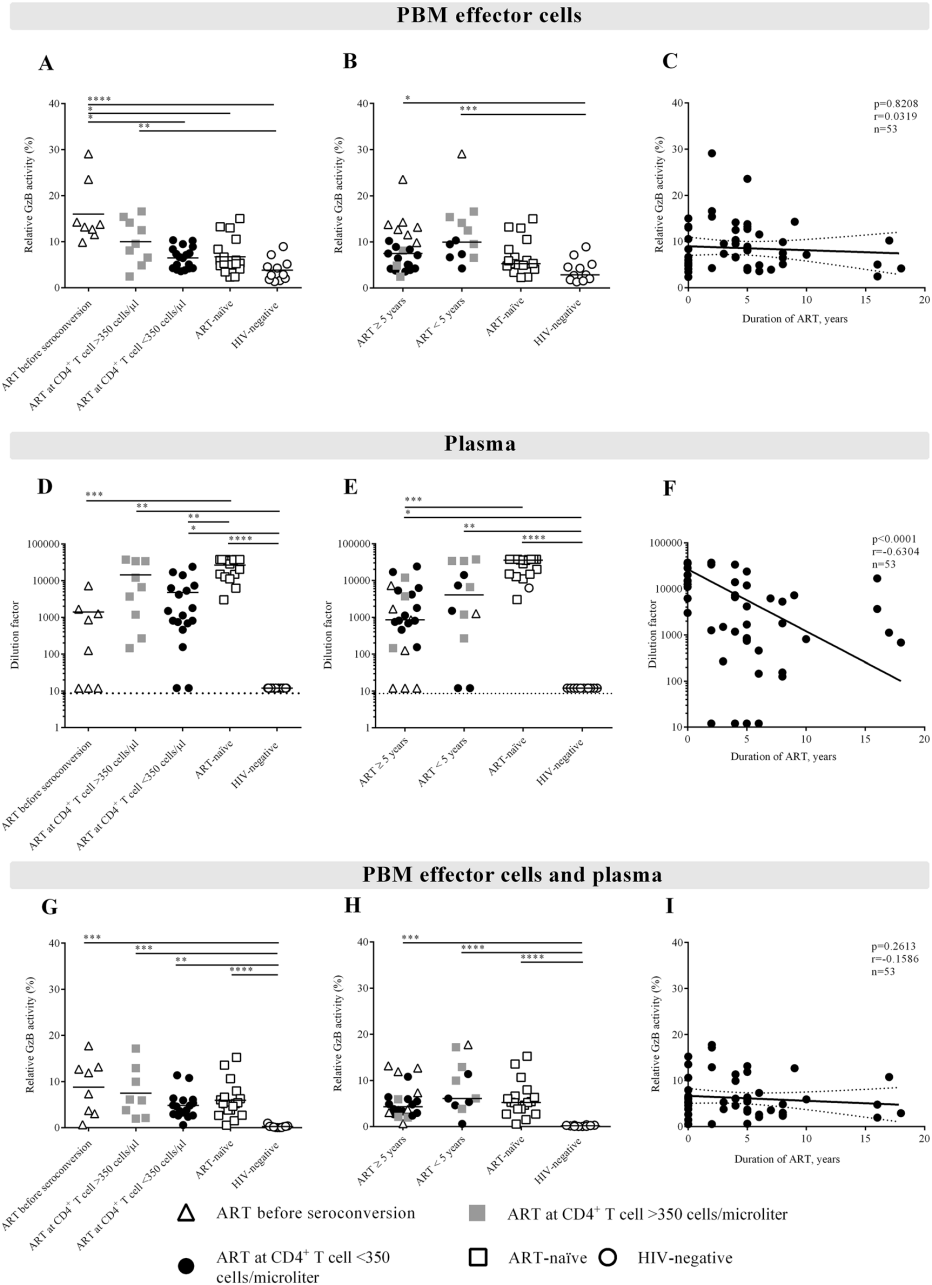
Increased ADCC-mediated activity in the PBM effector cells. The individuals in the study started on ART either before seroconversion (triangles), at CD4^+^ T cell counts above 350 cells/μl blood (gray quadrants), at CD4^+^ T cell counts below 350 cells/μl blood (filled circles), or were either ART-naïve (open quadrants) or HIV-negative controls (open circles). The ADCC mediated by the PBM effector cells was defined as the highest percentage of GzB activity after background subtraction at a given dilution. A) PBM effector cells are mediating significantly higher ADCC in individuals treated before seroconversion compared to individuals initiating ART at CD4^+^ T cell counts below 350 cells/μl blood, ART-naïve individuals and to the HIV-negative control group. B) Seven out of the 8 individuals treated before seroconversion have been in treatment for 5 years or more. C) The duration of ART in years in correlation to the relative percentage of GzB activity for the PBM effector cells. D) The ADCC activity mediated by plasma from individuals treated before seroconversion could be significantly less diluted compared to ART-naïve individuals. This was also the case for individuals who had initiated ART at CD4^+^ T cell counts above 350 cells/μl blood. E) The ADCC activity mediated by plasma from individuals who were treated could be significantly less diluted compared to the ART-naïve group. F) A significant inverse correlation was observed between the dilution factor and the duration of ART in years. G) Combining individuals’ PBM effector cells and plasma showed no significant difference between the HIV-positive individuals independent of when ART was initiated. H-I) The ADCC activity of PBM effector cells and plasma was independent of the duration of ART in years. In A-C and G-I GzB activity was normalized to the frequency of NK cells in each individual, in order to evaluate the activity per NK cell. The statistical significance is indicated in each Figure. Linear regression curves with 95-confidence intervals are indicated in C and I and non-linear regression curve in F. Not significant (NS) mean a p≥0.05; * means 0.01<p<0.05; ** means 0.001<p<0.01; *** means 0.0001<p<0.001 and; **** means p<0.0001.

The cut-off value (>8.64) was determined by the mean background of the 617 HIV-negative samples plus three standard deviations. The background was subtracted for each measurement. The ADCC levels were normalized to the positive control to overcome day-to-day variation in the assay and displayed as relative Granzyme B (GzB) activity as either percentages or dilution factors (plasma). When evaluating the ability of individuals’ PBM effector cells to mediate ADCC in combination with HIVIG (Fig 1A–1C) or in combination with the individuals’ plasma (Fig 1G–1I) we normalized the GzB activity to the frequency of CD16^pos^ NK cells. The ADCC value for each individual’s plasma was defined as the highest dilution factor above the cut-off value. The ADCC value mediated by the PBMCs and in the autologous model was defined as the highest relative GzB activity in percentages at a certain dilution. For titration curves see S1 Fig.

### Staining of NK cells receptors

Either whole blood or PBMCs were stained. The difference in receptor expression on NK cells in whole blood and thawed PBMCs was initially established. We found a significant difference in the frequency of CD16^pos^ cells expressing CCR7, CD57 and CD70 when using thawed cells compared to whole blood (data not shown). No difference was seen in the frequency of CD3^-^ cells expressing CD16. For this reason, we only perform receptor analysis on CD16^pos^ cell on whole blood (n = 43). For analysis comparing the frequency of CD16^pos^ expression, PBMCs and whole blood was used (n = 53). Either 1x10^6^ thawed PBMCs or 100 μl of whole blood were incubated for 20 minutes at room temperature in the dark and in the presence of LIVE/DEAD^®^ Fixable Dead Cell Stain Kit marker (Life Technologies, Naerum, Denmark), anti-CD3 PerCP (BD Biosciences), anti-CD8 Qdot605 (Life Technologies), anti-CD56 AlexaFluor488 (eBioscience, San Diego, CA), anti-CD16 APC-H7 (BD Biosciences), anti-NKp46 PECy7 (BD Biosciences), anti-CD57 APC (BD Biosciences), anti-CD27 AlexaFluor700 (eBioscience), anti-CCR7 PerCP/Cy5.5 (BioLegend, London, United Kingdom) and anti-CD70 PE (BD Biosciences) antibodies.

The antibodies were pre-titrated to determine the optimal staining concentration. Whole blood was lysed for 20 minutes at room temperature using BD FACS Lysing Solution (BD Biosciences). The cells were then washed and fixed using BD Stabilizing Fixative (BD Biosciences). The fluorescence levels on the stained cells were acquired using BD LSRII and analyzed using FlowJo (Ashland, OR, Treestar version 8.8.7). For detailed gating strategies, see S2 Fig. The NK cells were defined as CD3^neg^CD56^pos/neg^CD16^pos^. The total NK cell percentages and NK cell subsets are expressed as a percentage of the total lymphocytes. The number of NK cells (i.e. number of cells per blood volume) was calculated from the NK cell percentages of total lymphocytes as determined by whole blood staining followed by flow cytometry and the lymphocyte count shown in Table 1.

### Anti-HIV gp120 enzyme-linked immunosorbent assay (ELISA)

The anti-gp120-specific IgG subtype titers were determined by ELISA, as previously described [31]. The protein used for coating was, if possible, matched to the individuals’ HIV-1 subtype (Table 1). Plasma from individuals with either an unknown HIV-1 subtype or with either subtype G or C was tested against subtype B. The proteins used were the same as used in the ADCC assay, as described above. The data were read and illustrated as the absorbance at equivalent plasma dilutions (S3 Fig). The assay was modified by the use of four different conjugates: mouse-anti human IgG1, IgG2, IgG3 and IgG4 (Life Technologies).

### Statistical analysis

The data analysis was performed using GraphPad Prism 6 software version 6.0c (GraphPad Software Inc., La Jolla, CA) and the medians and inter quartile ranges were calculated. All data were tested for normality. The differences between the groups were tested using a non-parametric (Dunn’s multiple comparisons and Kruskal-Wallis test). The Mann-Whitney two-tailed test was used to test difference between two groups. A non-parametric Spearman two-tailed test was used to test for correlations.

## Results

### An enhanced ability of NK effector cell to mediate ADCC is linked to the duration of ART

The ADCC activity was assessed by three different strategies. i) The ability of individuals’ PBM effector cells (as a source of NK cells) to mediate ADCC was tested using the individuals’ PBMCs and HIVIG. ii) The ability of the individuals’ antibodies to mediate ADCC was tested using the individuals’ plasma and healthy donor PBMCs. iii) The ability of the individuals’ antibodies and individuals’ PBM effector cells to mediate ADCC was tested in an autologous model.

The ADCC activity of the PBMCs was higher in individuals treated with ART before seroconversion compared to either individuals initiating ART at a low CD4^+^ T cell count (<350 cells/μl blood), ART-naïve individuals or the HIV-negative control group (Fig 1A). The higher ADCC response of the PBMCs in the group treated before seroconversion was not explained by the number of years that these individuals had received ART, even though seven out of the 8 individuals in this group had received ART for 5 years or more (Fig 1B). The individuals, who had been on ART for 5 years or more, independent of when ART was initiated, did not mediate higher ADCC compared to the individuals who had been treated for less than 5 years or the ART-naïve individuals (Fig 1B). In contrast, the individuals on ART deviated from the HIV-negative control group (Fig 1B). No direct correlation was found between the duration of ART and relative GzB activity (Fig 1C). In these analysis the GzB activity was normalized to the frequency of NK cells in each individual, in order to evaluate the activity per NK cell rather than an increased proportion of NK cells.

We found a reduction in the ability of antibodies to mediate ADCC (i.e., the plasma) in individuals treated before seroconversion compared to ART-naïve individuals (p<0.001, Fig 1D). This finding may be explained by the fact that 7 of these 8 individuals had received ART for several years (Fig 1E). The individuals who initiated ART at CD4^+^ T cell counts below 350 cells/μl blood also deviated from the ART-naïve group (p<0.01, Fig 1D). There was no difference between the ART-naïve group and the individuals who had initiated ART at CD4^+^ T cell counts above 350 cells/μl blood (Fig 1D). This result may be influenced by the duration of treatment as 6 out of the 9 individuals who initiated ART at CD4^+^ T cell counts above 350 cells/μl blood had been on ART for less than 5 years (Fig 1E). We found an inverse correlation (p<0.0001) between the plasma dilution factor and the number of years of ART (Fig 1F), emphasizing that the duration of treatment reduces the ADCC activity of plasma antibodies. Excluding individuals that was non-B subtype did not change the results.

In the autologous model system, the ability of the PBM effector cells and the plasma to mediate ADCC in combination reflected that improved NK cell activity due to ART initiation was negatively affected by the decreased ability of plasma antibodies to mediate ADCC (Fig 1G–1I). There was also no correlation between the relative GzB activity and the duration of ART.

### Long-term ART increases the frequency of CD16^pos^ NK cells and correlates with ADCC activity

**Fig 2:**
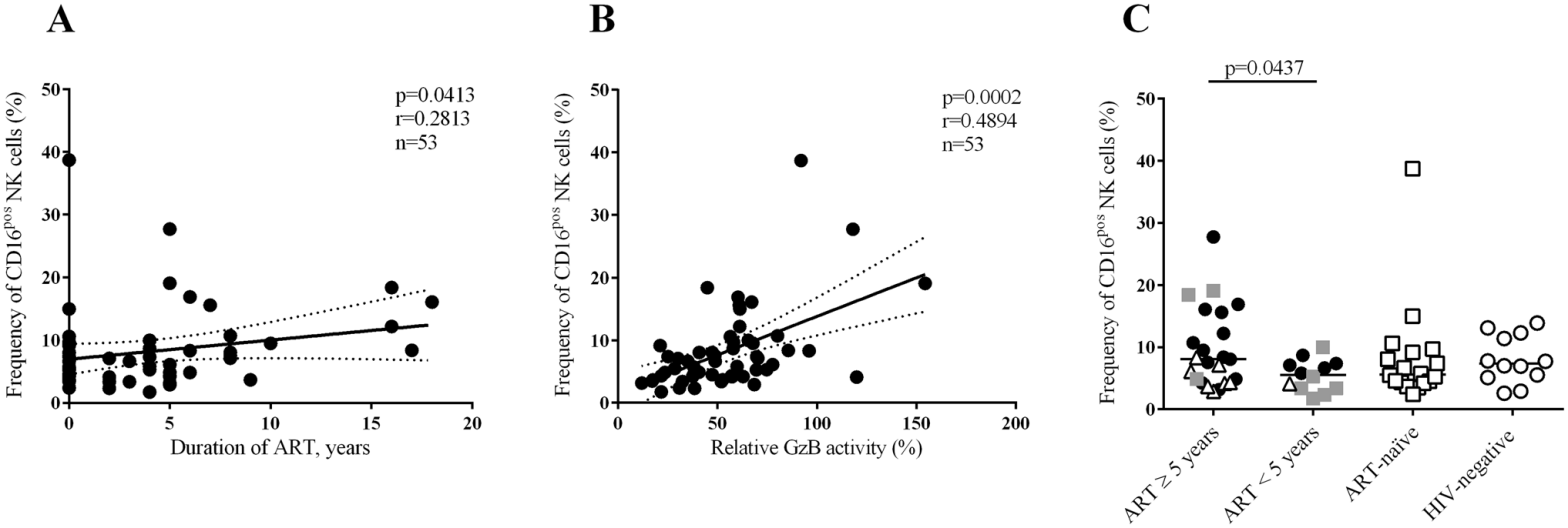
The frequency of CD16^pos^ NK cells increases with the duration of ART. A-B) The frequency of CD16^pos^ NK cells correlates with the duration of ART in years and with relative GzB activity. The total NK cell percentages and NK cell subsets are expressed as a percentage of the total lymphocytes. Linear regression curves with 95-confidence intervals are indicated. C) The individuals who had been treated for 5 years or more had significantly increased frequencies of CD16^pos^ NK cells compared to the individuals who had been treated for less than 5 years.

There was no correlation between PBM effector cell-mediated ADCC activity and either the CD4^+^ T cell counts or the viral load (data not shown). Moreover, immune activation, as measured by CRP and IL-6 (Table 1), did not differ between the groups (data not shown). However, we observed a correlation between the frequency of CD16^pos^ NK cells and the duration of ART (p = 0.0413) and between the frequency of CD16^pos^ NK cells and the relative GzB activity (p = 0.0002) (Fig 2A and 2B). The individuals who had been treated with ART for 5 years or more had a higher frequency of CD16^pos^ NK cells compared to individuals who had been treated with ART for less than 5 years (p = 0.0437, Fig 2C). The relative low r values indicates that other factors may influence the correlation e.g. the high inter-patient differences that is also seen for the HIV uninfected individuals.

The mean fluorescence intensity of CD16^pos^ NK cells did not correlate to either the duration of ART or the relative GzB activity mediated by PBM effector cells (data not shown). The frequency of the CD56^dim^CD16^pos^ NK cells, a subpopulation of the CD16^pos^ NK cells, also correlated with the duration of ART (p = 0.0190, data not shown) and the relative GzB activity mediated by PBM effector cells (p<0.0001, data not shown). The number of CD16^pos^ NK cells and the duration of ART did not correlate with one another. Moreover, there was no correlation between the number of CD16^pos^ NK cells and the relative GzB activity mediated by PBM effector cells (data not shown).

### The ADCC activity of the NK effector cells correlates with the expression of the lymphoid homing marker CCR7

**Fig 3:**
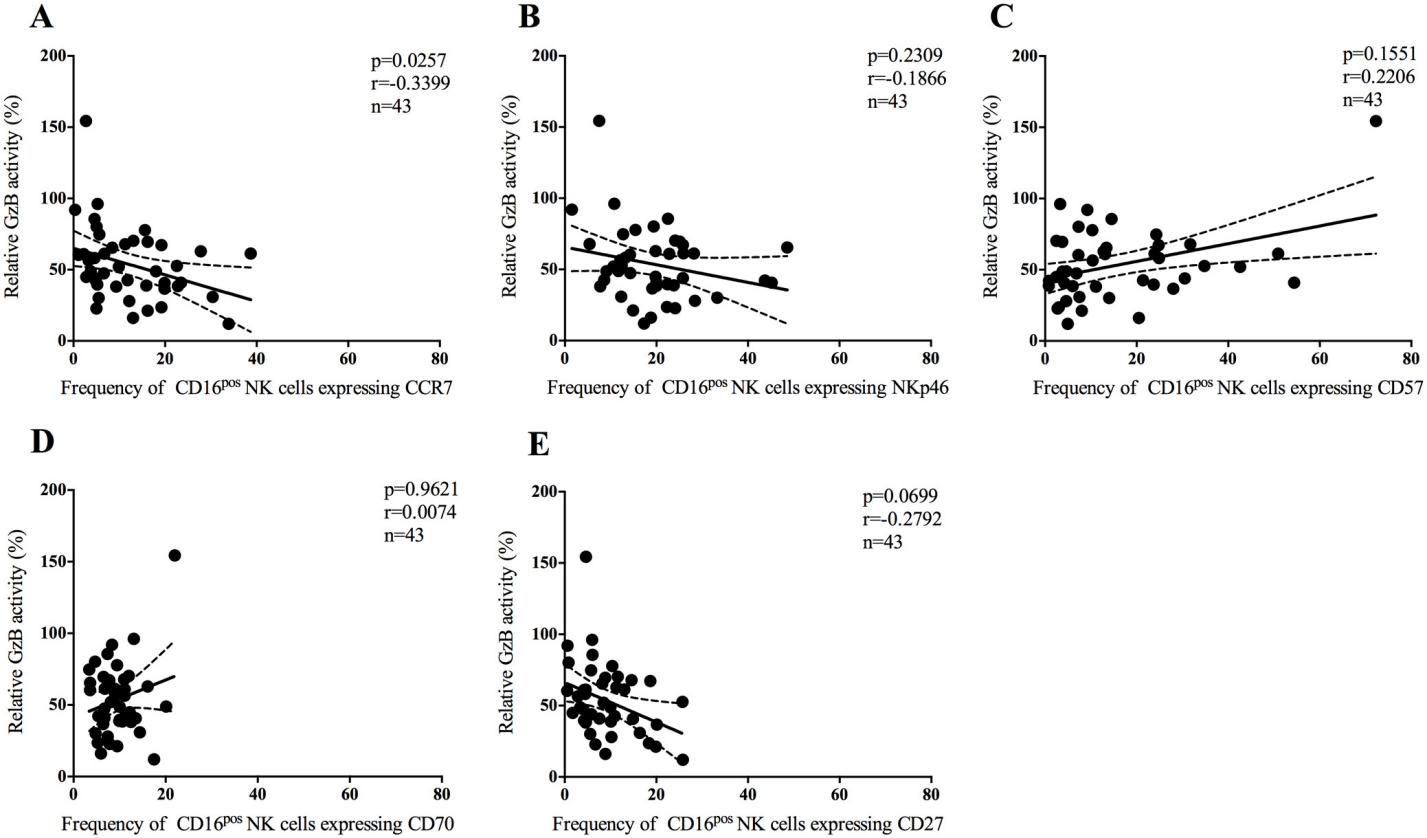
The frequency of CD16^pos^ NK cells expressing CCR7 correlates with the relative percentage of GzB activity. Correlation between the relative GzB activity mediated by PBM effector cells to the five phenotypic NK markers CCR7, NKp46, CD57, CD70 and CD27. A) The frequency of CD16^pos^ NK cells expressing CCR7 correlated inversely with relative GzB activity. B-E) No correlation was found between the frequency of CD16^pos^ NK cells expressing NKp46, CD57, CD70 and the relative GzB activity. The total NK cell percentages and NK cell subsets are expressed as a percentage of the total lymphocytes. Linear regression curves with 95-confidence intervals are indicated in each Figure.

We investigated the frequency of CD16^pos^ NK cells expressing CCR7, NKp46, CD57, CD70 or CD27 using whole blood and correlated this to the ADCC activity mediated by PBM effector cells. The frequency of CD16^pos^ NK cells expressing CCR7 was inversely correlated to the relative GzB activity mediated by PBM effector cells (p = 0.0257, Fig 3A). There was no correlation between the frequency of CD16^pos^ NK cells expressing NKp46, CD57, CD70 and CD27 and the relative GzB activity mediated by PBM effector cells (p = 0.2309, p = 0.1551, p = 0.9621 and p = 0.0699, Fig 3B–3E, respectively).

We correlated the levels of the five NK cell markers to the duration of ART. There was no correlation between the frequencies of CD16^pos^ NK cells expressing either of the markers. Moreover, no difference was found in the expression levels of either CCR7, NKp46, CD57, CD70 or CD27 between individuals who initiated ART before seroconversion, at CD4^+^ T cell counts above 350 cells/μl blood, at CD4^+^ T cell counts below 350 cells/μl blood and ART-naïve individuals.

## Discussion

In this study, we evaluated ADCC activity in HIV-1-positive individuals who initiated ART at different stages of disease: before seroconversion, at CD4^+^ T cell counts above 350 cells/μl blood and at CD4^+^ T cell counts below 350 cells/μl blood (at a relevant immunodeficiency). The ability of the NK cells to mediate ADCC was significantly improved in individuals who had been treated before seroconversion compared to the individuals initiating ART at CD4^+^ T cell counts below 350 cells/μl blood and the ART-naïve individuals. This finding did not appear to be biased by the length of time that individuals had received ART. In a longitudinal study, we have previously shown that NK cell mediated ADCC improves after only 6 months of ART [13]. However, in this study, treated individuals did not have improved NK cell function compared to ART-naïve individuals. Inter-individual differences make it more difficult to show a difference when combining groups in a cross-sectional study compared to an analysis of intra-individual changes.

Although, we did not see a direct correlation between the ability of PBM effector cells to mediate ADCC and the duration of ART we found a significant correlation between the frequency of NK cells expressing CD16 and the duration of ART. The frequency of NK cells expressing CD16 also correlated to the ADCC activity mediated by the PBM effector cells. Interestingly, we could not show the same difference when comparing treated versus ART-naïve individuals. The frequency of NK cells expressing CD16 is significantly higher in individuals who have been treated with ART for 5 years or more compared to individuals who have been treated with ART for less than 5 years. Four of the 18 ART-naïve individuals had plasma viral loads below 300 copies/ml (Table 1), nevertheless these individuals had a relatively low GzB activity mediated by plasma and by PBM effector cells, which did not diminish the difference between treated and ART-naïve individuals.

We explored whether the PBMC effector function increased during ART due to factors such as changes in the NK cell phenotype. An inverse correlation was found between the frequency of CD16^pos^ NK cells expressing the lymphoid homing marker CCR7 and GzB activity, confirming the results from a recent study [13]. Reduced surface expression levels of NKp46 have been associated with impaired cytolytic function in viremic HIV-infected individuals [28] but the receptor expression is partially restored after successful ART [32]. A recent study showed that activation of NK cells via CD16 by anti-HIV antibodies reduces surface expression of NKp46 [27]. Here, no correlation could be demonstrated between the frequency of NK cells expressing NKp46 and the cytotoxic function of the NK cells. The frequency of NK cells expressing CD57 could not be correlated to the ADCC activity even though CD57 is linked to the maturation of NK cells [22, 23] and is acquired on CD56^dim^ NK cells upon activation. Moreover, CD57^pos^ NK cells are more responsive to signaling through the CD16 receptor than CD57^neg^ NK cells [23]. However, no direct correlation between ADCC mediated by NK cells and CD57 has previous been shown and was not demonstrated here. The frequency of NK cells expressing CD70 and CD27 did not correlate with GzB activity. The CD70 receptor has been linked to NK cell dysfunction and is up regulated during HIV infection but restored to normal levels during highly active antiretroviral therapy [21]. The CD27 receptor is mainly expressed on CD56^bright^ NK cells, which are poorly cytotoxic [33] and CD70 is a ligand of the CD27 receptor.

Our phenotypic analysis was performed on whole blood as we found a surprisingly large difference in receptor expression when comparing the whole blood and PBMC assays. This deviation between marker expression on NK cells from separated PBMC and whole blood has previously been found on CD8^+^ T cells [34–36]. For this reason only whole blood was used for the analysis of the frequency of CD16^pos^ cells expression of the receptors of interest.

Although NK function improves during ART, the ability of plasma antibodies to mediate ADCC decreases. This was also observed in another study [14] and is believed to be due to the lack of antigen stimulation. We found the anti-gp120 antibody titers of IgG1 were significantly lower in individuals who have been undergoing ART for either 5 years or more or for less than 5 years compared to ART-naïve individuals (p<0.0001 and p<0.01, respectively, S3A Fig); the anti-gp120 antibody titers of IgG3 showed the same trend (p<0.01 and p<0.05, respectively, S3C Fig). The IgG2 and IgG4 titers were not decreased in the individuals receiving ART compared to ART-naïve individuals (S3B and S3D Fig, respectively). It has been shown that the plasma antibodies in individuals who are able to control their viral loads mediate higher and broader ADCC responses [37–40]. The specificity of the ADCC-mediating antibodies was not assessed, however it would be interesting to examine if the specificity was different in individuals treated before seroconversion compared to individuals treated after seroconversion. This should be undertaken in another study.

We performed a comprehensive analysis of NK cells alone, plasma antibodies alone, and plasma antibodies in combination with effector cells in an autologous model. Other studies have demonstrated that NK cells in HIV-infected individuals secrete significantly higher levels of GzB than NK cells in healthy individuals, [15] despite the various defects in NK cell function caused by HIV [18, 41]. This finding suggests that NK cells might be pre-activated by binding to the Fc region of HIV-specific antibodies *in vivo* [15]. This model, which combines NK cells and autologous plasma, may be the most physiologically relevant system to study the actual impact of ADCC *in vivo*. However, the enhanced NK cell activity does not seem to compensate for the decrease in ADCC mediating antibodies during ART.

Some of the correlations reported in our study are limited by the R-values assisting the significant p-values, of course this should be considered when interpreting the results. It is possible that increasing the number of studied individuals could improve the R-values for the spearman correlations.

In summary, this study suggests that individuals receiving ART before seroconversion improves NK cell cytotoxic function, as measured by ADCC, while the levels of ADCC mediating antibodies decrease during ART. Moreover, the NK phenotype characterized by low levels of CCR7 and correlated with higher levels of ADCC activity. It might be therapeutically beneficial to combine ART and vaccines that induce ADCC mediating antibodies as part of a cure strategy. A boost of antibodies at a time when the NK cell function is restored may together give a competent ADCC able to eliminate infected cells expressing HIV-1 antigens.

## Supporting Information

S1 Fig The titration curves for PBM effector cells, plasma alone, and PBM effector cells together with plasma. A) The titration curves for the PBM effector cells mediating ADCC. B) The titration curves for plasma antibodies mediating ADCC. C) The titration curves for PBM effector cells and plasma antibodies mediating ADCC.
(PDF)

S2 Fig Gating strategy for NK cell receptor expression. The cells were initially gated on a forward scatter area (FSC-A) versus height (FSC-H) plot to exclude doublets from the analysis. The lymphocytes were identified in a side scatter area (SSC-A) versus FSC-A plot. The dead cells were confirmed to be V450 bright and excluded in an SSC-A versus V450 plot. CD3-negative cells were identified, and NK cells were gated for the three subsets: CD56^pos^CD16^pos^, CD56^dim^CD16^pos^ and CD56^neg^CD16^pos^. Finally, the frequency of NK cells expressing CCR7, CD27, CD57, CD70 and NKp46 was identified in the CD56^pos^CD16^pos^, CD56^dim^CD16^pos^ and CD56^neg^CD16^pos^ NK cell subsets.
(PDF)

S3 Fig The IgG1 and IgG3 anti-gp120 titers are down-regulated during ART. The anti-gp120 antibody binding titers of IgG1 (diluted 1:1000), IgG2 (diluted 1:10), IgG3 (diluted 1:100) and IgG4 (diluted 1:10) were measured. The data were read and illustrated as absorbance values. A) A significant decrease was observed in the titers of IgG1 in individuals treated for 5 years or more (white diamonds) and for less than 5 years (black diamonds) compared to the ART-naïve (white square) (p<0.0001 and p<0.01, respectively). B) No difference in IgG2 antibody titer was observed between the treated and ART-naïve individuals. C) A significant decrease was observed in the IgG3 titers in individuals who had been treated for 5 years or more and in individuals who had been treated for less than 5 years compared to the ART-naïve (p<0.01 and p<0.05, respectively). D) There was no significant difference in IgG4 titers between the treated individuals and ART-naïve individuals. Not significant (NS) means p≥0.05; * means 0.01<p<0.05; ** means 0.001<p<0.01; and **** means p<0.0001.
(PDF)
