# Supplementary figures and images for: HIV-Specific Antibody-Dependent Cellular Cytotoxicity (ADCC) -Mediating Antibodies Decline while NK Cell Function Increases during Antiretroviral Therapy (ART)
Source: PLoS One. 2015 Dec 22;10(12):e0145249. doi: 10.1371/journal.pone.0145249 (PMC4692281; doi:10.1371/journal.pone.0145249)

Supporting Information 1

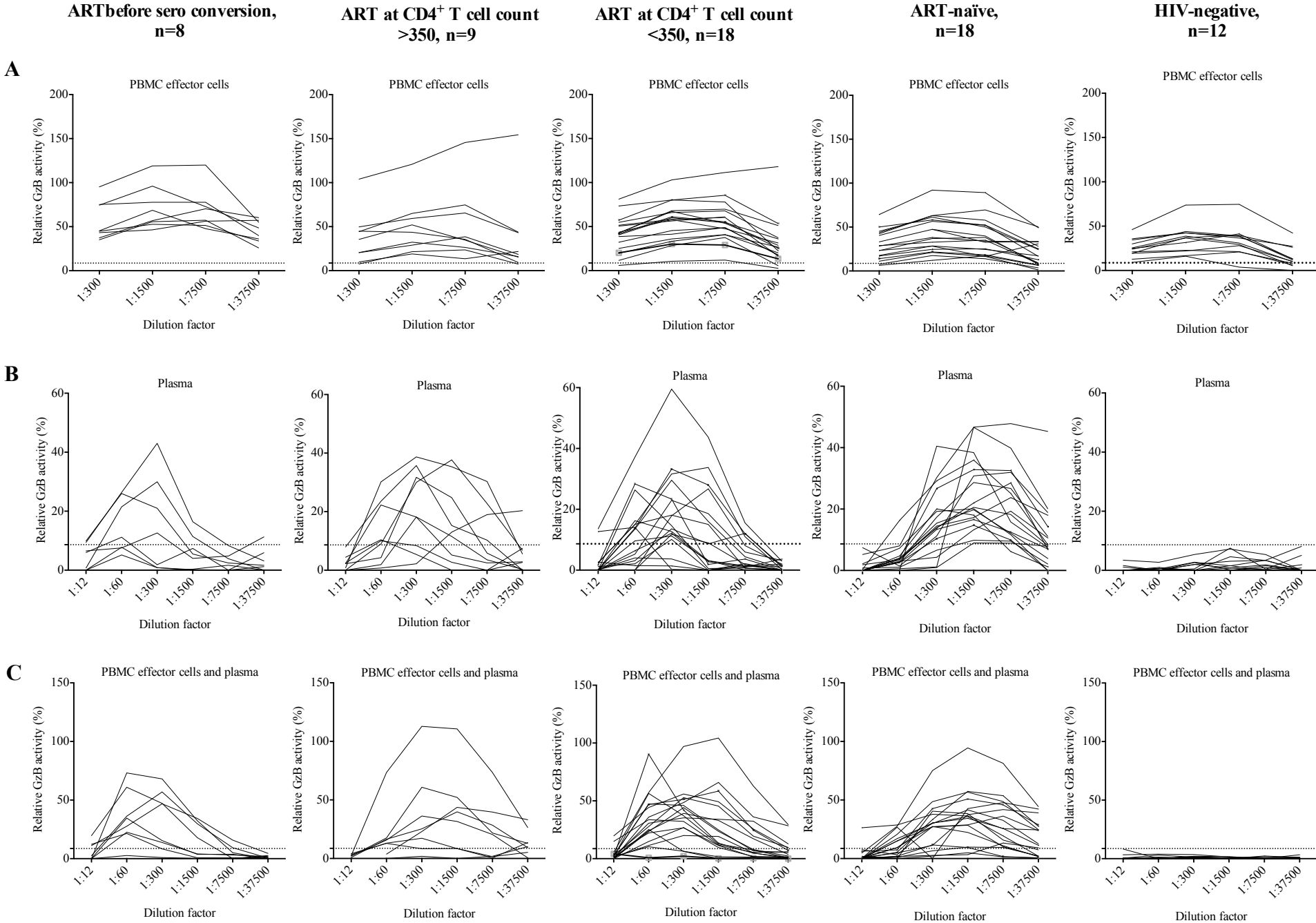

Supplement: S1 Fig — A) The titration curves for the PBM effector cells mediating ADCC. B) The titration curves for plasma antibodies mediating ADCC. C) The titration curves for PBM effector cells and plasma antibodies mediating ADCC. (PDF) [file pone.0145249.s001.pdf]

# Supporting Information 2

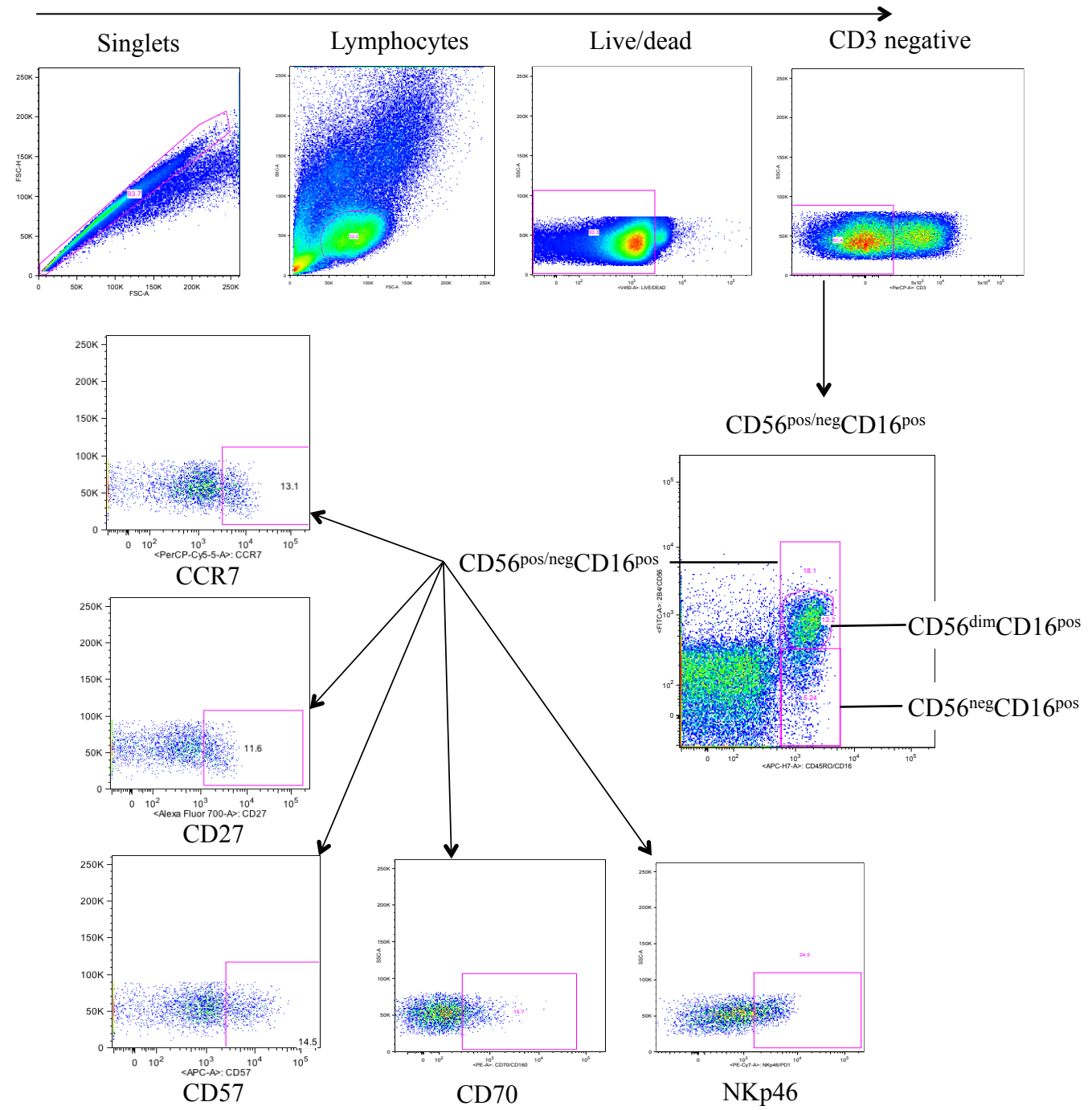

Supplement: S2 Fig — The cells were initially gated on a forward scatter area (FSC-A) versus height (FSC-H) plot to exclude doublets from the analysis. The lymphocytes were identified in a side scatter area (SSC-A) versus FSC-A plot. The dead cells were confirmed to be V450 bright and excluded in an SSC-A versus V450 plot. CD3-negative cells were identified, and NK cells were gated for the three subsets: CD56posCD16pos, CD56dimCD16pos and CD56negCD16pos. Finally, the frequency of NK cells expressing CCR7, CD27, CD57, CD70 and NKp46 was identified in the CD56posCD16pos, CD56dimCD16pos and CD56negCD16pos NK cell subsets. (PDF) [file pone.0145249.s002.pdf]

Supporting Information 3

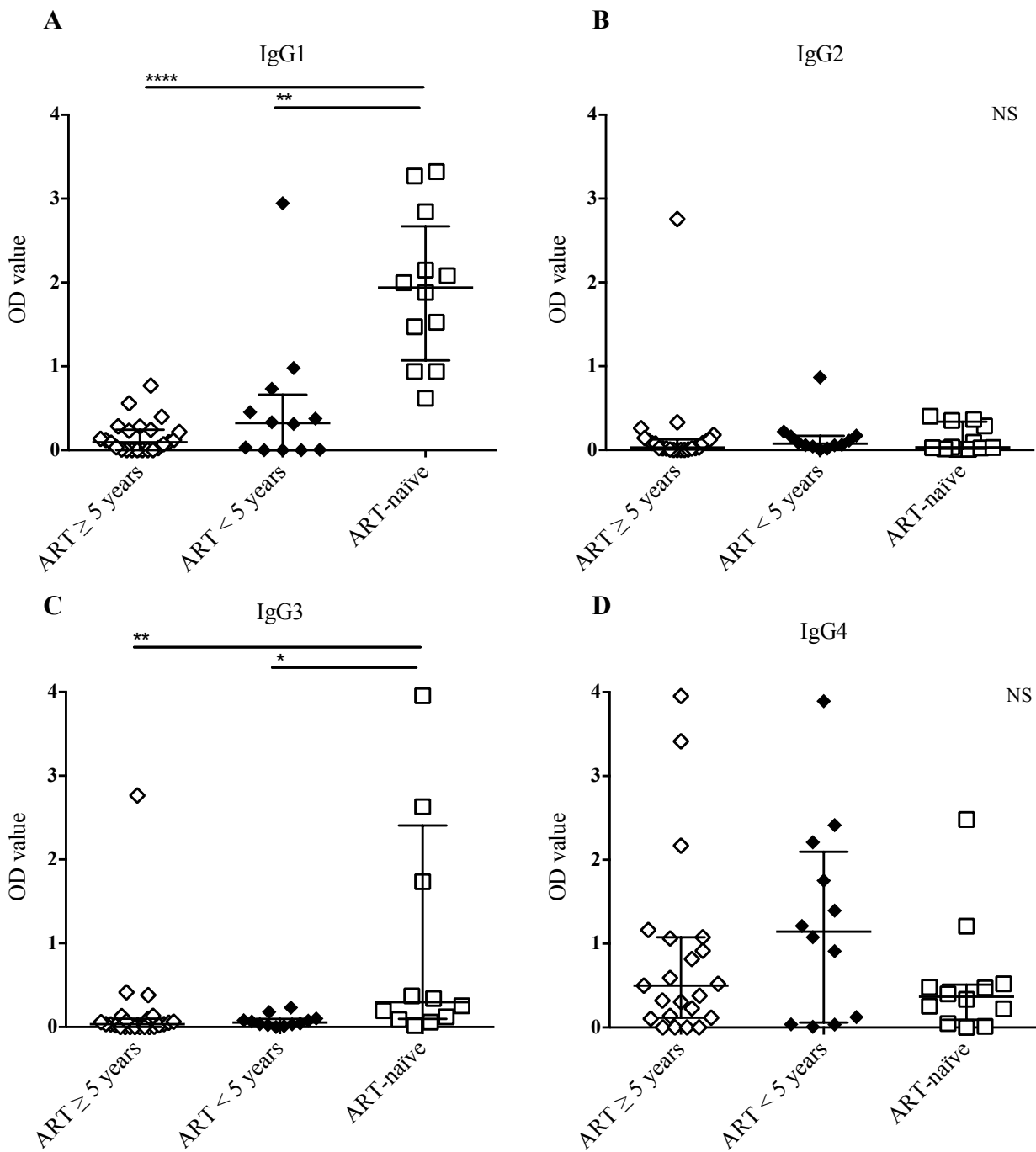

Supplement: S3 Fig — The anti-gp120 antibody binding titers of IgG1 (diluted 1:1000), IgG2 (diluted 1:10), IgG3 (diluted 1:100) and IgG4 (diluted 1:10) were measured. The data were read and illustrated as absorbance values. A) A significant decrease was observed in the titers of IgG1 in individuals treated for 5 years or more (white diamonds) and for less than 5 years (black diamonds) compared to the ART-naïve (white square) (p<0.0001 and p<0.01, respectively). B) No difference in IgG2 antibody titer was observed between the treated and ART-naïve individuals. C) A significant decrease was observed in the IgG3 titers in individuals who had been treated for 5 years or more and in individuals who had been treated for less than 5 years compared to the ART-naïve (p<0.01 and p<0.05, respectively). D) There was no significant difference in IgG4 titers between the treated individuals and ART-naïve individuals. Not significant (NS) means p≥0.05; * means 0.01<p<0.05; ** means 0.001<p<0.01; and **** means p<0.0001. (PDF) [file pone.0145249.s003.pdf]
